# A novel small molecule inhibitor of human Drp1

**DOI:** 10.1038/s41598-022-25464-z

**Published:** 2022-12-13

**Authors:** Ayeshah A. Rosdah, Belinda M. Abbott, Christopher G. Langendorf, Yali Deng, Jia Q. Truong, Helen M. M. Waddell, Naomi X. Y. Ling, William J. Smiles, Lea M. D. Delbridge, Guei-Sheung Liu, Jonathan S. Oakhill, Shiang Y. Lim, Jessica K. Holien

**Affiliations:** 1 St Vincent’s Institute of Medical Research, Fitzroy, VIC Australia; 2 Faculty of Medicine, Universitas Sriwijaya, Palembang, Indonesia; 3 Department of Surgery and Medicine, University of Melbourne, Melbourne, VIC Australia; 4 Department of Biochemistry and Chemistry, La Trobe Institute for Molecular Science, La Trobe University, Melbourne, VIC Australia; 5 Department of Anatomy and Physiology, The University of Melbourne, Parkville, VIC 3010 Australia; 6 Australian Catholic University, Fitzroy, VIC Australia; 7 Centre for Eye Research Australia, Royal Victorian Eye and Ear Hospital, East Melbourne, VIC Australia; 8 Drug Discovery Biology, Faculty of Pharmacy and Pharmaceutical Sciences, Monash University, Melbourne, VIC Australia; 9 National Heart Centre, National Heart Research Institute Singapore, Singapore, Singapore; 10 School of Science, RMIT University, GPO Box 2476, Melbourne, VIC 3001 Australia; 11 Menzies Institute for Medical Research, University of Tasmania, Hobart, TAS Australia

**Keywords:** Biochemistry, Cell biology, Drug discovery, Cardiology

## Abstract

Mitochondrial dynamin-related protein 1 (Drp1) is a large GTPase regulator of mitochondrial dynamics and is known to play an important role in numerous pathophysiological processes. Despite being the most widely used Drp1 inhibitor, the specificity of Mdivi-1 towards human Drp1 has not been definitively proven and there have been numerous issues reported with its use including off-target effects. In our hands Mdivi-1 showed varying binding affinities toward human Drp1, potentially impacted by compound aggregation. Herein, we sought to identify a novel small molecule inhibitor of Drp1. From an initial virtual screening, we identified DRP1i27 as a compound which directly bound to the human isoform 3 of Drp1 via surface plasmon resonance and microscale thermophoresis. Importantly, DRP1i27 was found to have a dose-dependent increase in the cellular networks of fused mitochondria but had no effect in Drp1 knock-out cells. Further analogues of this compound were identified and screened, though none displayed greater affinity to human Drp1 isoform 3 than DRP1i27. To date, this is the first small molecule inhibitor shown to directly bind to human Drp1.

## Introduction

Mitochondrial dynamin-related protein 1 (Drp1) is a large GTPase regulator of mitochondrial fission that plays a central role in various signalling cascades including cellular metabolism, cell proliferation and cell death^1–4^. Others have also reported non-canonical roles of Drp1, such as in peroxisome division^5–7^, vesicle endocytosis in neurons^8^ and maintenance of cardiac mitochondrial respiration via modulation of mitochondrial permeability transition pore^9^.

The quinazoline derivative, Mdivi-1, is the most frequently studied reversible allosteric inhibitor of Drp1. This small molecule has been shown to inhibit the GTPase activity of Dnm1, a yeast homolog of Drp1, with an IC_50_ of 1–10 μM^10^. Several in vitro and in vivo studies have demonstrated the cytoprotective effect of Mdivi-1 in various cell types and injury models such as ischaemia–reperfusion injury, doxorubicin-induced cytotoxicity and propofol-induced cell death^11^. These preclinical findings suggest the promising therapeutic potential of Drp1 inhibition. However, the specificity of Mdivi-1 towards human Drp1 has been called into question.

The seminal work by Cassidy et al.^10^ showed that 50 μM of Mdivi-1 could effectively inhibit staurosporin-induced mitochondrial fission in mammalian COS cells. Conversely, a recent study by Bordt et al.^12^ could not replicate this finding in COS-7 cells with similar experimental settings. In fact, they reported that Mdivi-1 poorly inhibited the GTPase activity of human Drp1 with an inhibitory constant (Ki) of > 1.2 mM^12^. Instead, Bordt et al.^12^ demonstrated that Mdivi-1 is a weak inhibitor of mitochondrial complex I in primary rat cortical neurons as well as in both the Drp1 wild-type and knockout (KO) mouse embryonic fibroblasts (MEFs), suggesting a Drp1-independent effect. They also suggested that Mdivi-1 is likely to have multiple cellular targets that are independent of Drp1 due to its thiophenol molecular structure.

Various studies have also revealed Drp1-independent protective mechanisms by Mdivi-1, such as direct inhibition of rapidly activating delayed rectifier potassium channels (Ikr) in HL-1 cells^13^ and mitochondrial complex I in MEFs^12^, as well as repressing Neuregulin 1 (Nrg1), a transcriptional factor for yeast-to-hyphae morphogenesis, in *C. albicans*^14^.

Altogether, the lack of specificity of Mdivi-1 towards human Drp1 may have contributed to the contradictory findings of Mdivi-1 in some experiments reporting a lack of cytoprotective effect and increased cell death with Mdivi-1^15–20^. Herein, we sought to find an alternative Drp1 inhibitors which bound directly to Drp1, inhibited its GTPase activity and had a cellular effect on mitochondrial dynamics.

## Methods and materials

### In silico screening

OpenEye software was used to conduct a virtual screen of compound libraries against the X-ray crystal structure of human Drp1 (PDB code: 4H1V). Virtual compound libraries were assembled from online databases of a number of commercial suppliers such as ChemBridge. Prior to screening, the libraries were refined to select only “drug-like” compounds using FILTER (OpenEye, NM, USA) giving ~ 6.5 million structures, which were then converted to three-dimensional (3D) structures using Omega^21^. Virtual screening was carried out with FRED^22^ using the ChemGauss scoring function. The highest scoring and most diverse 50 compounds were purchased and assayed. A subsequent 2D similarity search of a hit compound was performed using Unity (Tripos, MO, USA) and twenty-six compound analogues with high 2D complementarity (Tanimoto coefficient > 0.80) were purchased for assay.

### Protein expression and purification

The recombinant human Drp1 protein (His-tagged human Drp1 isoform 3, kindly provided by Professor Michael Ryan, Monash University, Australia) was generated as described previously^23^. Briefly, cDNA for human Drp1 (isoform 3) was cloned into pET21b vector and transformed in Rosetta (DE3) competent cells (Novagen, MA, Merck Millipore). Transformed cells were propagated in Luria–Bertani broth and protein expression was initiated by the addition of 0.5 mM isopropyl β-D-1-thiogalactopyranoside. Cells were harvested by centrifugation at 3,500 g for 20 min at 4 °C, and the pellet was re-suspended in lysis buffer containing 50 mM Tris HCl (pH 7.3), 500 mM NaCl, 50 mM imidazole, 2.5 mM β-mercaptoethanol, 0.01 mM LEUPEP, 0.1 mM AEBSF and 1 mM benzaminidium chloride. The cells were lysed using a pre-cooled EmulsiFlex-C5 homogenizer (Avestin, Ontario, Canada) on ice and clarified by centrifugation at 17,000 g for 30 min. The clarified cell lysate was loaded onto a 5 mL Nickel Chelating Sepharose Fast Flow column (GE Healthcare, Buckinghamshire, UK) and Drp1 protein was eluted with wash buffer containing 50 mM Tris–HCl (pH 7.6), 150 mM NaCl, 2.5 mM β-mercaptoethanol and 400 mM imidazole. Eluted proteins were equilibrated with a buffer containing 50 mM Tris–HCl (pH 7.6), 150 mM NaCl, 10% glycerol and 2 mM TCEP using a PD-10 desalting column (GE Healthcare).

### Surface Plasmon Resonance (SPR)

All SPR experiments were performed on a Biacore T200 instrument (GE Healthcare Life Sciences, Uppsala, Sweden) at 25 °C in the presence of HEPES buffered saline containing 20 mM phosphate (pH 7.5), 137 mM NaCl, 2.7 mM KCl and 0.05% Tween-20 (HBS + P). Protein was tethered to a CM5 chip (GE Life Sciences, Germany) using an amine coupling method in sodium acetate buffer (pH 5) and protein running buffer containing 20 mM HEPES, 160 mM NaCl, 100 uM EDTA and 0.005% Tween-20. Sensor surface was activated with 1:1 mixture of 0.4 M EDC and 0.1 M NHS for 7 min, followed by incremental injections of the protein to reach ~ 8000 RU (flow rate 10 μL/minute) for a calculated maximum analyte binding capacity (Rmax) of 30–50 RU. Following attachment, sensor surface was deactivated with 1 M ethanolamine (pH 8.5) and washed with running buffer. The tethered protein was left to stabilise at least for 1 h in HBS + P before compound injection.

Compounds were dissolved in 100% dimethyl sulfoxide (DMSO) and then added to HBS + P at a final DMSO concentration of 2%. Prior to conducting compound SPR, a DMSO solvent correction was performed following the Biacore Laboratory guideline 29-0057-18 (GE Healthcare Life Sciences, Buckinghamshire, UK). Compounds were injected at 30 μL/minute for 60 s followed by 400 s dissociation time, across the chip in a threefold dose–response manner from a highest concentration of 500 µM (100 µM for Mdivi-1). SPR results were analysed using the Biacore T200 Evaluation Software v.1.

### Microscale thermophoresis (MST)

Compound binding was assessed in against the human Drp1 isoform-3 protein using MST Monolith NT.115 instrument (NanoTemper Technologies, Mȕnchen, Germany). Protein was labelled using the NanoTemper Protein Labeling Kit RED-NHS Monolith NT.115 (Amine Reactive) (NanoTemper Technologies) and diluted to final concentration of 20 nM. The following instrument settings were applied: medium MST power and LED power was on auto-detect. Compounds were serially diluted two-fold in MST buffer (phosphate buffered solution (PBS) pH 7.4, 150 mM NaCl, 0.05% Tween-20), ensuring a final concentration of 5% DMSO. Samples were centrifigued for 10 min at 15,000 rpm before adding to Monolith premium capillaries (NanoTemper Technologies). Concentration range of compounds were as follows: 0.015–500 µM of Mdivi-1, 0.076 µM–2.5 mM of DRP1i27 and 0.15 µM–5 mM of DRP1i27i and DRP1i27g.

### Dynamic light scattering (DLS)

Identification of compound aggregation threshold was performed using DLS Zetasizer Nano ZS (Malvern Pananalytical, Worcestershire, UK). 100 µL of samples were measured in low-volume plastic micro cuvettes (BrandTech Scientific, CT, USA) at 25 °C using default size measurement settings. Buffers (PBS pH 7.4, 150 mM NaCl containing 2% or 5% DMSO with or without 0.05% Tween-20) were filtered through a 0.2 µm syringe filter prior to compound dilution. Mdivi-1 and DRP1i27 were serially diluted three-fold from 500 µM until the volume distribution of particle size was similar to that of the DMSO control. To specifically determine aggregation threshold, further dilution was carried out between concentrations where aggregation was first observed.

### Drp1 GTPase activity

The GTPase activity of Drp1, which is required for the latter to mediate mitochondrial fission, was examined using the colorimetric GTPase assay kit (Innova Biosciences, Cambridge, UK) according to the manufacturer’s instructions^23^. Compounds were diluted in DMSO (all with a final concentration of 0.1% DMSO). Recombinant human Drp1 protein isoform-3 (final concentration 0.25 ng/µL) was diluted in assay buffer containing 50 mM Tris HCl (pH 7.5) and 2.5 mM MgCl_2_, followed by incubation with compounds for 15 min at 37 °C in a 96 well plate. 0.5 mM GTP, diluted in assay buffer, was then added into the mixture followed by incubation at 37 °C for 30 min.

Upon binding to Drp1, GTP is hydrolysed into GDP and free phosphate (Pi)^24^. Therefore, the degree of GTPase inhibition was assessed by measuring the concentration of Pi released into the assay buffer, which is detected by a malachite green-based dye. The intensity of Pi-dye complex was assessed by measuring the light absorbance at 635 nm wavelength using a microplate reader (BenchMark Plus, MS, USA). Absorbance of each experimental group was normalised to the vehicle control (DMSO).

### Simulated ischaemia–reperfusion injury

HL-1 cells were plated in 24-well plate pre-coated with 5 μg/mL human fibronectin (Merck Millipore, MA, USA) in 0.02% porcine gelatin solution (Sigma-Aldrich, MA, USA). Cells were maintained in Claycomb media (Sigma-Aldrich) supplemented with 0.1 mM (±)-norepinephrine (+)-bitartrate salt (Sigma-Aldrich), 2 mM GlutaMAX (Thermo Fisher Scientific, MA, USA), 100 μg/mL penicillin/streptomycin (Thermo Fisher Scientific) and 10% non-heat inactivated foetal bovine serum (FBS) (Bovogen Biologicals, Victoria, Australia). The simulated ischaemia–reperfusion injury was performed as described in Rosdah et al.^25^. To simulate ischaemia, cells were cultured in ischaemic buffer (25 mM NaHEPES, 1 mM KH_2_PO_4_, 10 mM NaHCO_3_, 74 mM NaCl, 1.2 mM MgCl_2_.6H_2_O, 16 mM KCl, 1.2 mM CaCl_2_, 10 mM 2-deoxy-d-glucose) and hypoxia (< 0.1% O_2_) was induced in an airtight GENBox anaerobic chamber containing a Genbox Anaer generator sachet (BioMérieux, Marcy l’Etoile, France) at 37 °C. After 5 h of simulated ischaemia, simulated reperfusion was performed in DMEM low glucose media (5 mM glucose) supplemented with 10% FBS and 100 μg/mL penicillin/streptomycin for 1 h at 37 °C in a humidified 5% CO_2_ incubator. HL-1 cells were treated with either DRP1i27 (5, 10 or 50 µM) or 0.1% DMSO vehicle control during simulated ischaemia and reperfusion. Cell death was determined by staining with 1 μg/mL propidium iodide and 3 μg/mL Hoechst 33258. The number of dead cells (propidium iodide positive) was counted and expressed as a percentage over total number of cells (Hoechst 33258 positive). At least 100 cells were counted per group for each independent experiment.

### Hydrogen peroxide-induced oxidative stress

Human foreskin fibroblasts (HFF-1, ATCC, VA, USA) were treated with 3 mM hydrogen peroxide plus vehicle control (0.1% DMSO), Mdivi-1 (50 µM) or individual compound at 50 µM. After 2.5–3 h of incubation at 37 °C in a humidified 5% CO_2_ incubator, cells were stained with 1 μg/mL propidium iodide and 3 μg/mL Hoechst 33258 to determine the percentage of dead cells. Images were captured at 20 × objective with an inverted microscope (Olympus IX71). The number of dead cells (propodium iodide positive) was counted and expressed as a percentage over total number of cells (Hoechst 33258 positive). At least 100 cells were counted per group for each independent experiment.

### Doxorubicin-induced cardiomyocyte injury

Cardiomyocytes were derived from human induced pluripotent stem cells (iPSCs, CERA007c6 cell line) as previously described with modifications^26,27^. Briefly, human iPSCs were seeded onto Matrigel (Corning) coated plates at a density of 1.25 × 10^5^ cells/cm^2^ in TeSR-E8 medium supplemented with 10 μM Y−27632 (Abcam). After 48 h (cells ~ 90% confluent) (day 0), medium was replaced with RPMI-1640 basal medium (Thermo Fisher Scientific) containing B-27 without insulin supplement (Thermo Fisher Scientific), growth factor reduced Matrigel (1:60 dilution) and 10 μM CHIR99021 (Cayman Chemical). At day 1, medium was replaced with RPMI 1640 basal medium containing B-27 without insulin supplement. At day 2, medium was changed to RPMI 1640 basal medium containing B-27 without insulin supplement and 5 μM IWP-2 (Sigma-Aldrich) for 72 h. From day 5, cells were cultured in cardiomyocyte medium containing RPMI 1640 basal medium, B-27 supplement (Thermo Fisher Scientific) and 200 μg/mL L-ascorbic acid 2-phosphate sesquimagnesium salt hydrate (Sigma-Aldrich). At day 12, differentiated cardiomyocytes were dissociated into single cells and split 1:2 onto Matrigel coated plates in DMEM/F-12 GlutaMAX medium supplemented with 20% FBS (Sigma-Aldrich), 0.1 mM 2-mercaptoethanol, 0.1 mM nonessential amino acids, 50 U/mL penicillin/streptomycin and 10 μM Y−27632. At day 13, medium was changed to cardiomyocyte medium. From days 14–19, cardiomyocytes were enriched by culturing in glucose-free DMEM medium (Thermo Fisher Scientific) supplemented with 4 mM lactate (Sigma-Aldrich).

Enriched cardiomyocytes were replated onto Matrigel-coated 48-well plates at a cell density of 5 × 10^4^ cells/cm^2^ for 2 days. Cardiomyocytes were then treated with vehicle control DMSO (0.1%), DRP1i1 (50 μM), doxorubicin (5 μM) or combination of DRP1i1 and doxorubicin for 2 days. Release of lactate dehydrogenase (LDH) from cells can be used as an indicator of cellular damage. LDH levels in the conditioned media were assessed using a colorimetric CyQUANT LDH Cytotoxicity Assay Kit (Thermo Fisher Scientific) according to manufacturer’s protocol. Briefly, 50 μL of conditioned medium from each sample (in duplicate) was transferred to a 96-well flat bottom plate and 50 μL of Reaction Mixture was transferred to each well. Following the incubation at room temperature for 30 min in the dark, 50 μL of Stop Solution was added and absorbance was measured at 490 nm and 680 nm on a microplate spectrophotometer (Bio-Rad). All readings were then corrected for the background signal from the culture media and expressed as fold change.

### Mitochondrial morphology analysis

Human foreskin fibroblasts and Drp1 wildtype and KO MEF (kindly provided by Professor Hiromi Sesaki, Johns Hopkins University, USA)^28,29^ were transduced with pAS2.EYFP.puro lentiviruses (RNAiCore, Academia Sinica, Taiwan) carrying the mitochondrial-targeted EGFP sequence as described in Kalkhoran et al.^30^. Cells were then treated with DRP1i27 (5, 10 or 50 µM) or 0.1% DMSO vehicle control for 24 h.

Mitochondrial morphology in HL-1 cells were visualised by immunostaining of mitochondrial Hsp60 antibody (Abcam, MA, USA). Images were acquired using Olympus BX60 fluorescence microscope at 600 × magnification and analysed using Fiji imaging software (ImageJ v1.53c). Cells were categorised into predominantly (> 50%) having fused or fragmented mitochondria. At least 100 cells were analysed for each treatment group.

### Molecular docking

The human GTPase-GED fusion Drp1 crystal structure (PDB code 4H1V) was used with GOLD v5.7.1 (Cambridge Crystallographic Data Centre (CCDC), UK) docking software. The protein was protonated and its co-crystallised ligand, GNP, was extracted. All waters were removed in the docking run with toggle and trans-spin settings enabled. The selection of the extracted and included waters was based on the following criteria: (**1**) distance cut-off < 2 Å of any atom of GNP and < 3 Å of any residue atom; (**2**) water molecules not directly involved in binding of GNP and/or GNP and protein were excluded^31^.

The binding site was defined by selecting protein atoms within 6 Å of GNP, and the cavity was restricted to only solvent-accessible surfaces. All H-bond donors/acceptors were treated as solvent accessible. The chemical structures of DRP1i27 and the analogues were sketched in MarvinSketch v19.7 (ChemAxon, Hungary) and the lowest energy conformer for each ligand was selected. All compounds (including the control compound GNP) were docked using the default genetic algorithm with medium speed and ranked according to the ChemPLP scoring function. Early termination was not allowed. Full ligand flexibility was selected, everything else was kept as default.

A visual clustering analysis was conducted on all of the 10 possible ligand binding orientations per compound, with only orientations showing a strong clustering score (> 50%) deemed likely docking poses. The highest scoring (ChemPLP) compound from this cluster was utlised as the preferred ligand binding orientation. Protein–ligand interactions were calculated in PyMOL v2.3.2 (Schrodinger, USA) and LigPlot v1.4.5^32^.

### Statistics

All values are expressed as mean ± standard error of the mean (SEM). Significance of the differences was evaluated using one-way ANOVA followed by multiple comparison post-hoc analysis where appropriate. *p* ≤ 0.05 was considered statistically significant.

## Results

### Mdivi-1 shows inconclusive binding to human Drp1

Mdivi-1 was obtained from a screen of compounds which inhibited yeast Drp1 GTPase activity^10^. Considering < 50% sequence identity between yeast and human Drp1 (Supplementary Fig. 1), any functional effect exerted by Mdivi-1 in mammalian model must be interpreted cautiously as it may be independent of a direct interaction with human Drp1.

**Figure 1:**
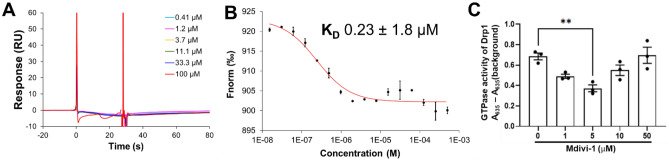
Binding affinity and Drp1 GTPase analyses of Mdivi-1. Representative (**A**) SPR and (**B**) MST results of Mdivi-1 (n = 2). (**C**) Mdivi-1 significantly inhibited Drp1 GTPase activity (n = 3). Data are expressed as mean ± SEM. **p ≤ 0.01 by one-way ANOVA with Bonferroni post hoc test.

In the SPR assay, no binding response was observed between Mdiv-1 and human isoform 3 Drp1, even at concentrations as high as 100 µM (Fig. 1A), suggesting that Mdivi-1 does not directly bind to human Drp1. However, Mdivi-1 showed a dose-dependent binding to human isoform 3 Drp1 in the MST assay with a K_D_ value of 0.23 ± 1.8 µM (Fig. 1B). Interestingly, Mdivi-1 exerted significant inhibition of GTPase activity of human Drp1 at 5 µM but this effect was not observed with increasing concentration of Mdivi-1 (Fig. 1C).

**Figure 2:**
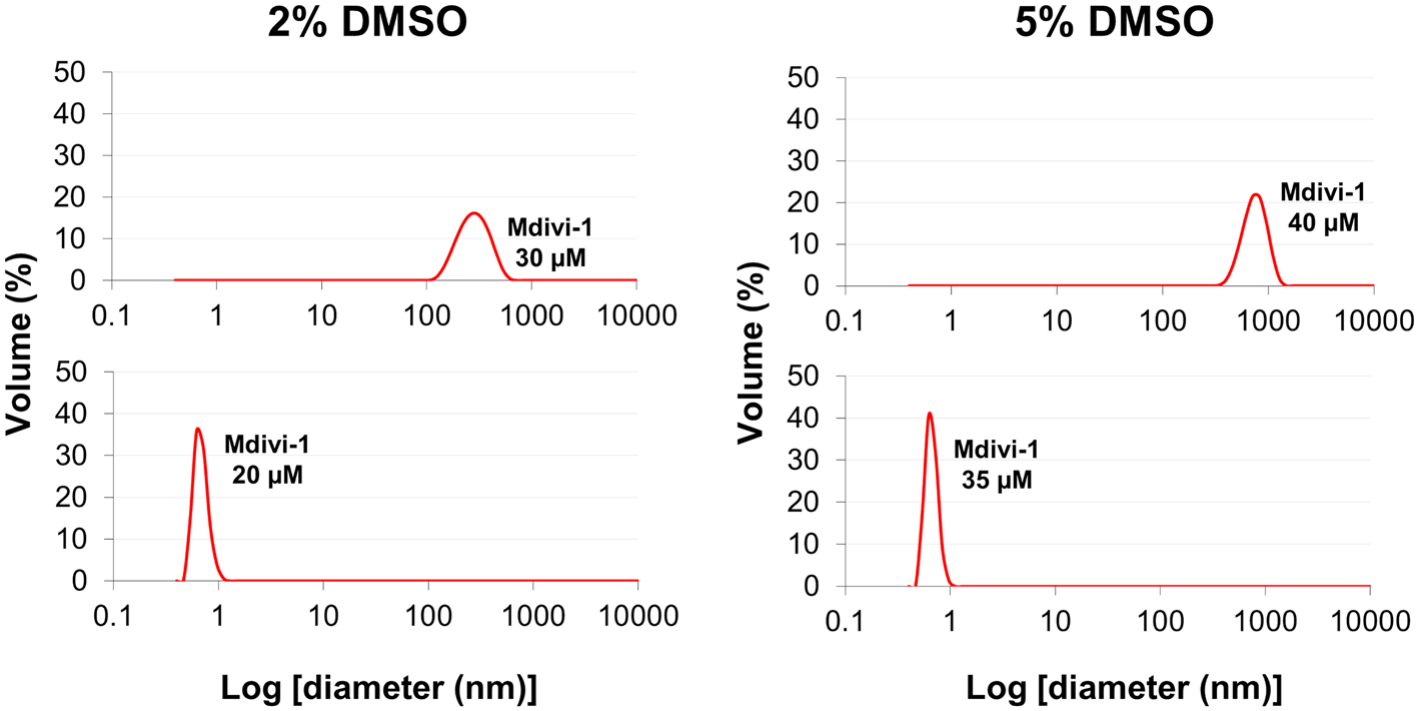
Representative dynamic light scattering for Mdivi-1. The particle size of Mdivi-1 increased from 20 µM to 30 µM in PBS with 2% DMSO (left panel) and from 35 to 40 µM in PBS with 5% DMSO (right panel). This mirrors the experimental conditions in both the SPR and MST assays.

This disparity between assays, combined with an MST-derived binding affinity of greater than tenfold the reported IC_50_ of Mdivi-1 (1–10 μM (Cassidy-Stone, et al., 2008)), prompted us to investigate whether the binding response observed in MST assay was associated with compound aggregation. Using DLS, Mdivi-1 was shown to aggregate at either 20 µM or 35 µM in PBS containing either 2% or 5% DMSO, respectively (Fig. 2 and Supplemental Fig. 2). Interestingly, the addition of 0.05% Tween-20 increased the aggregation threshold from 20 to 70 µM in 2% DMSO, but reduced this threshold from 35 to 18.5 µM in 5% DMSO (Supplemental Fig. 2). The presence of Tween micelles broadens the DLS peaks, with the predicted average diameter of Mdivi-1 aggregates range from 6–352 nm and 193–1558 nm with and without Tween, respectively. Although this wide range of aggregate size makes accurately defining the aggregation threshold of Mdivi-1 difficult, there is a clear shift in the diameter as the concentration of Mdivi-1 increases. These results suggest that any reported inhibition of Mdivi-1 at high concentration (18.5–70 µM, depending on solvent) should be interpreted with great caution.

### Identification of DRP1i27 as a novel inhibitor of human Drp1

**Figure 3:**
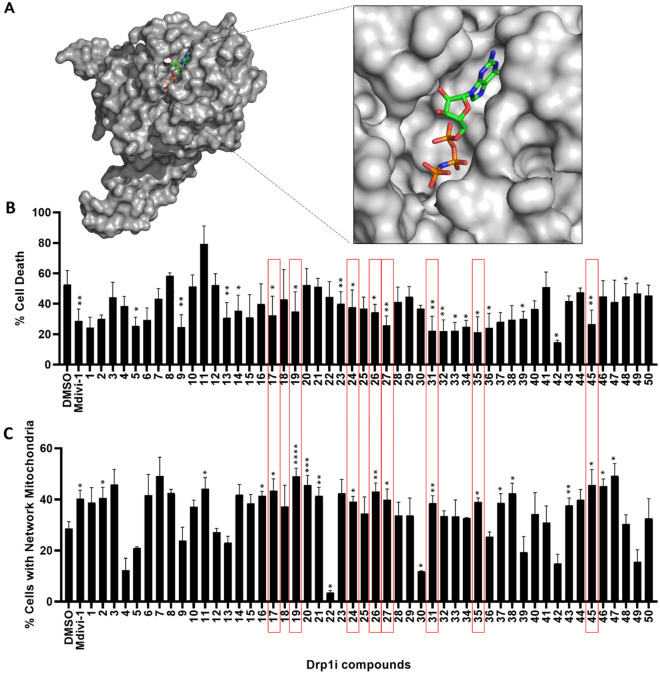
Identification of novel Drp1 inhibitors. (**A**) Crystal structure of Drp1 (4H1V, in gray) with its ligand, a non-hydrolysable analogue of guanosine triphosphate (in colour), used as a reference structure for molecular docking. Compound screening based on (**B**) inhibition of oxidative stress-induced cell death (n = 3) and (**C**) promotion of network mitochondrial morphology in human fibroblasts under basal condition (n = 4). Compounds which induced both a significant reduction in cell death induced by hydrogen peroxide treatment (**B**), and significant increase in percentage of cells with elongated mitochondria (**C**) are highlighted via a red box. Data are expressed as mean ± SEM. *p ≤ 0.05, **p ≤ 0.01, ***p ≤ 0.001, ****p ≤ 0.0001 by one-way ANOVA with Fisher’s LSD post hoc test.

In order to identify potent and drug-like inhibitors of human Drp1, we conducted an in silico screen using the Drp1 GTPase domain crystal structure (PDB code:4H1V)^33^. Screening procedures developed in-house with OpenEye Scientific software were used to search our in silico library of compounds for molecules with the capacity to bind to the putative GTP binding pocket of the Drp1 GTP-ase domain (Fig. 3A) and thus inhibit its function. We purchased the top 50 compounds from the in silico screen, based upon their calculated binding affinity and structural diversity. Eight of these compounds (DRPi17, DRPi19, DRPi24, DRPi26, DRPi27, DRPi31, DRP1i35 and DRPi45) induced both significant reduction in cell death and a significant increase in the percentage of cells with elongated mitochondria, when compared to DMSO control, indicating reduced Drp1 cellular activity (Fig. 3).

**Figure 4:**
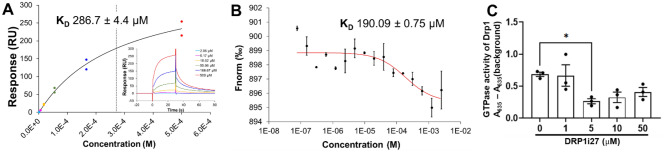
DRP1i27 directly binds to and inhibits GTPase activity of human Drp1. (**A**) Representative fitted and raw data of SPR and (**B**) MST showing binding response of DRP1i27 to human Drp1 isoform 3 (n = 2). (**C**) The effect of DRP1i27 on Drp1 GTPase activity (n = 3). Data are expressed as mean ± SEM. *p ≤ 0.05 vs DMSO by one-way ANOVA with Bonferroni post hoc test.

The eight most promising novel compounds were all screened for a direct interaction with human Drp1 via SPR, with one compound, DRP1i27, showing dose–response binding to human Drp1 isoform 3 with a K_D_ of 286 ± 4.4 µM (Fig. 4A). Follow-up via MST confirmed the binding with a K_D_ of 190.9 ± 0.75 µM (Fig. 4B). Furthermore, 5–50 µM of this compound was able to show a trend of inhibition of the conversion of GTP to GDP in the GTPase activity assay (Fig. 4C). However, a statistically significant reduction was only observed at 5 µM. DLS showed DRP1i27 has an aggregation threshold of 130 µM and 350 µM in 2% and 5% DMSO, respectively. The addition of 0.05% Tween-20 increased the aggregation threshold of DRP1i27 to at least 500 µM (Supplemental Fig. 2).

### DRP1i27 targets Drp1-mediated mitochondrial fission in cell line models and protects against simulated ischemia–reperfusion injury.

**Figure 5:**
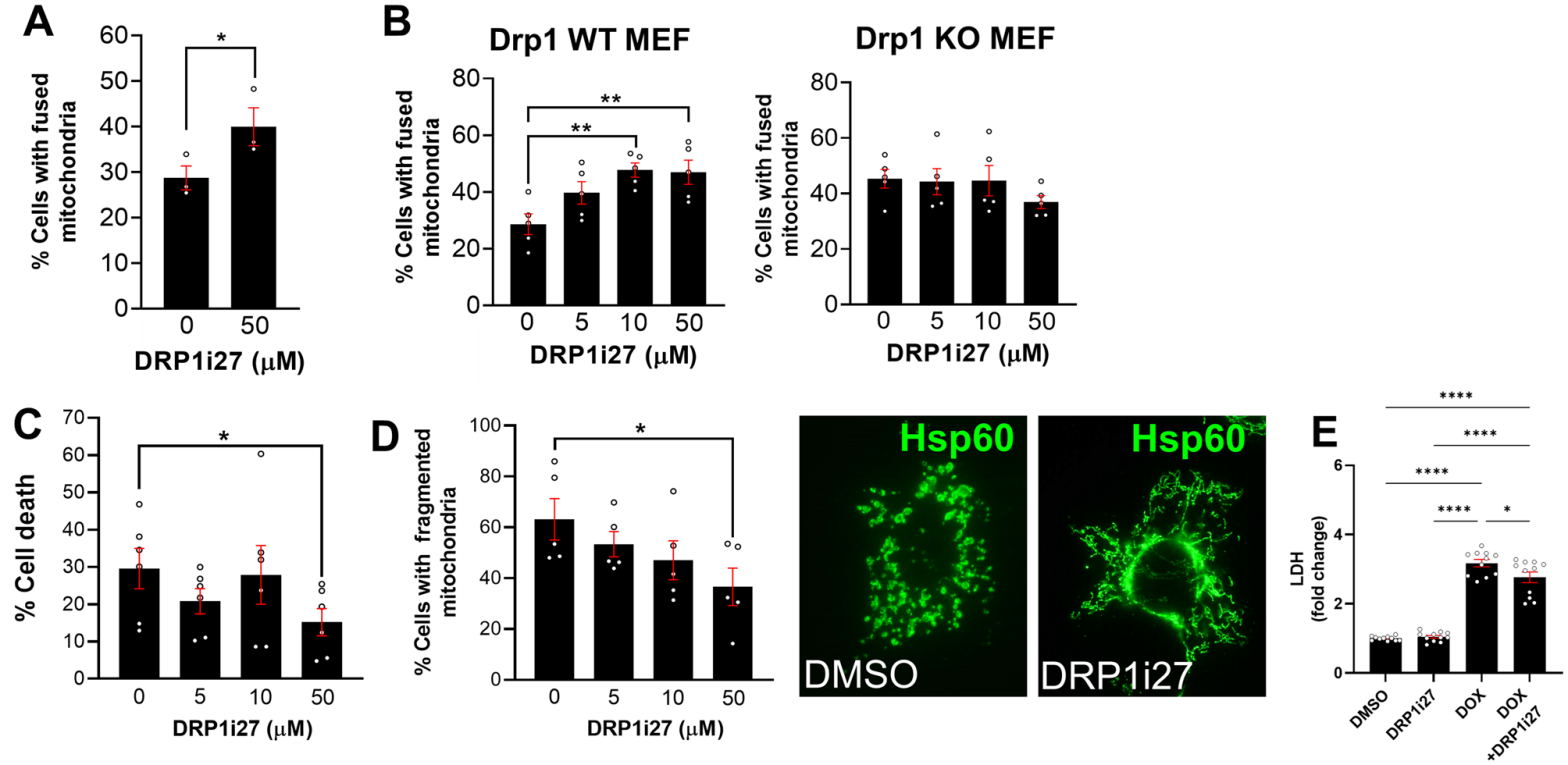
The effect of DRP1i1 on mitochondrial morphology and cell survival. **(A, B)** The effect of DRP1i27 on promoting mitochondrial fusion in **(A)** human fibroblasts as well as **(B)** Drp1 wild type and KO MEF (n = 3–5). **(C, D)** The effect of DRP1i27 on **(C)** cell death and **(D)** percentage of cells with fragmented mitochondria in HL-1 cells subjected to simulated-ischaemia–reperfusion injury (n = 5–6).**(E)** The effect of DRP1i27 on lactate dehydrogenase (LDH) production in human iPSC-derived cardiomyocytes treated with DMSO (0.1%), DRP1i27 (50 µM), doxorubicin (DOX, 5 µM) or doxorubicin +DRP1i27 in combination (n=11 from 3 independent experiments). Data are expressed as mean ± SEM. *p ≤ 0.05 vs DMSO by one-way ANOVA with Bonferroni post hoc test.

DRP1i27 was able to increase cellular networks of mitochondria in human and mouse fibroblasts (Fig. 5). Notably, a dose-dependent response was observed in Drp1 wild type MEFs, which reached statistical significance at 10 and 50 µM (Fig. 5B). Importantly, this compound had no significant effect on Drp1 KO MEFs, indicating a Drp1-dependent effect (Fig. 5B).

Murine atrial HL-1 cells treated with 50 µM of DRP1i27 during simulated ischaemia–reperfusion injury showed a significant reduction of cell death compared to the vehicle control (15.17 ± 3.67% in 50 µM DRP1i27 vs. 29.55 ± 5.45% in DMSO, *p* ≤ 0.05) (Fig. 5C). This was also accompanied by a significant reduction in percentage of cells with fragmented mitochondria at 50 µM DRP1i27 (36.62 ± 7.37% vs. 63.14 ± 8.12% in DMSO, *p* ≤ 0.05) (Fig. 5D).

To evaluate the cytoprotective effects of DRP1i27 against doxorubicin-induced toxicity, LDH release.

from human iPSC-derived cardiomyocytes was quantified. Treatment with DRP1i27 significantly reduced the cytotoxicity induced by 5 µM of doxorubicin (3.13 ± 0.16 vs. 3.41 ± 0.11 in doxorubicin, *P *< 0.05, Fig. 5E).

### Molecular Docking and compound analogues suggest a binding mode for DRP1i27

**Figure 6:**
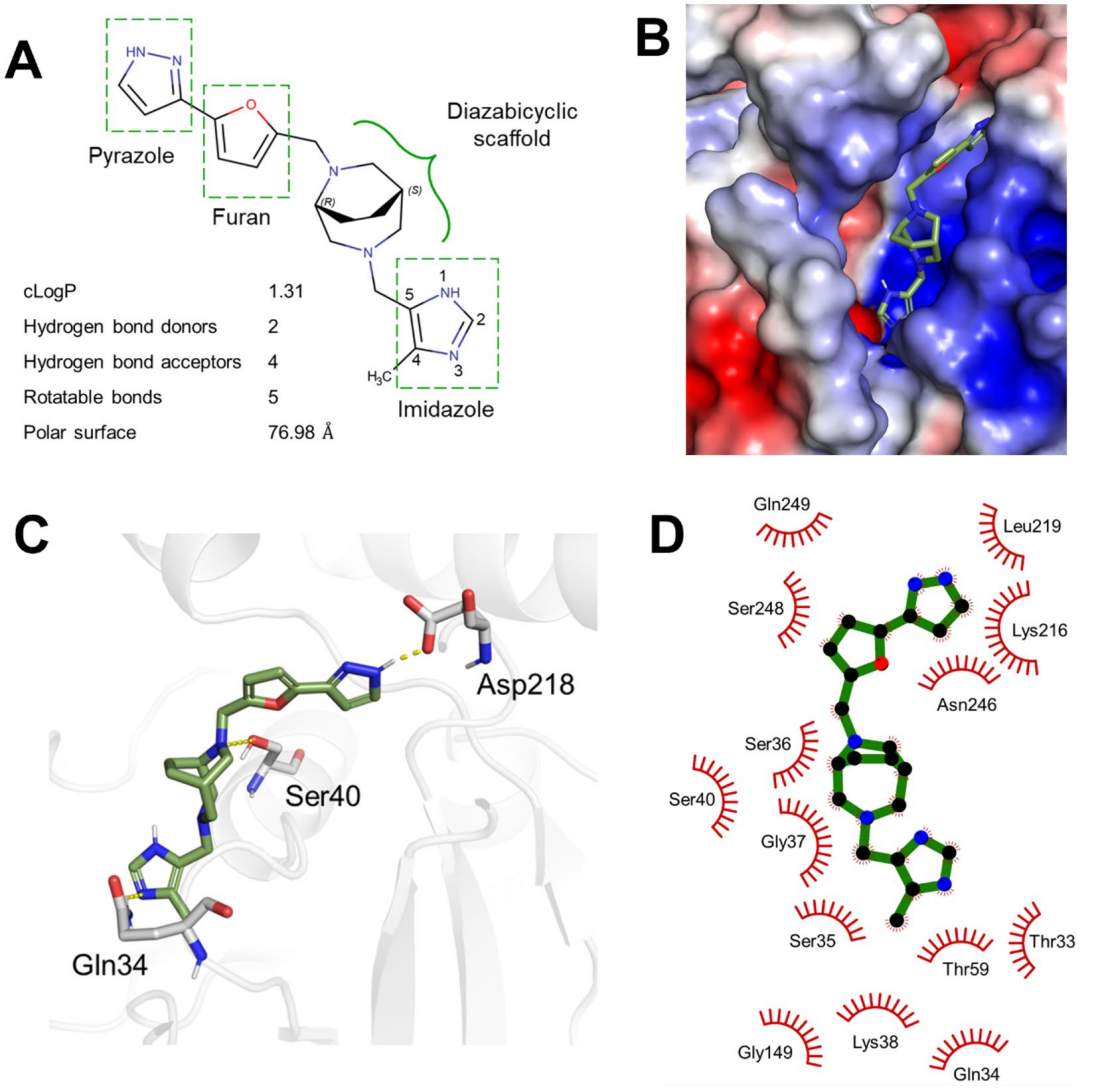
Molecular interactions of DRP1i27 at the active site of Drp1. **(A)** Chemical structure and physicochemical properties of DRP1i27. **(B)** Representative image of DRP1i27 in the active site of human Drp1 protein. Protein surface was coloured according to electrostatic potential of surface residues. Dark red, negative charge; Dark blue, positive charge. White, neutral charge. **(C)** 3D representation of hydrogen bonding interactions and **(D)** 2D representation of hydrophobic interactions of DRP1i27 at the active site. For clarity, non-polar hydrogen atoms are hidden from view. Secondary structure of the active site is depicted as light grey cartoon. Yellow dashed lines indicate hydrogen bonds. Blue, nitrogen atom; Red, oxygen atom. Lime green and gray stick backbones, carbon atom. Interactions were predicted using PyMOL v2.5.2 and LigPlot v1.4.5.

A molecular dock of DRP1i27 into the human Drp1 active site (PDB code: 4H1V) was conducted to assess how the compound was potentially interacting with Drp1. DRP1i27 contains a diazabicyclic scaffold with an imidazole at one end and a pyrazole connected to a furan at the other (Fig. 6A). Molecular docking showed that Drp1i27 is predicted to interact with the GTPase binding domain of Drp1 via putative polar interactions (Fig. 6B–C) and several hydrophobic interactions (Fig. 6D). Putative hydrogen bonds are formed between the amine group of the imidazole ring and the oxygen atom of the side chain of Gln34. One of the nitrogen atoms in the diazabicyclic ring is available to interact with the hydroxyl group of the side chain of Ser40. The amine group of the pyrazole ring also shows a putative hydrogen bond with the side chain of Asp 218 (Fig. 6C). In this orientation, there are proposed hydrophobic interactions of DRP1i27 with Thr33, Gln34, Ser35, Ser36, Gly37, Lys38, Ser40, Thr59, Gly149, Lys216, Leu219, Asn246, Ser248 and Gln249 of the GTPase binding domain of Drp1.

**Figure 7:**
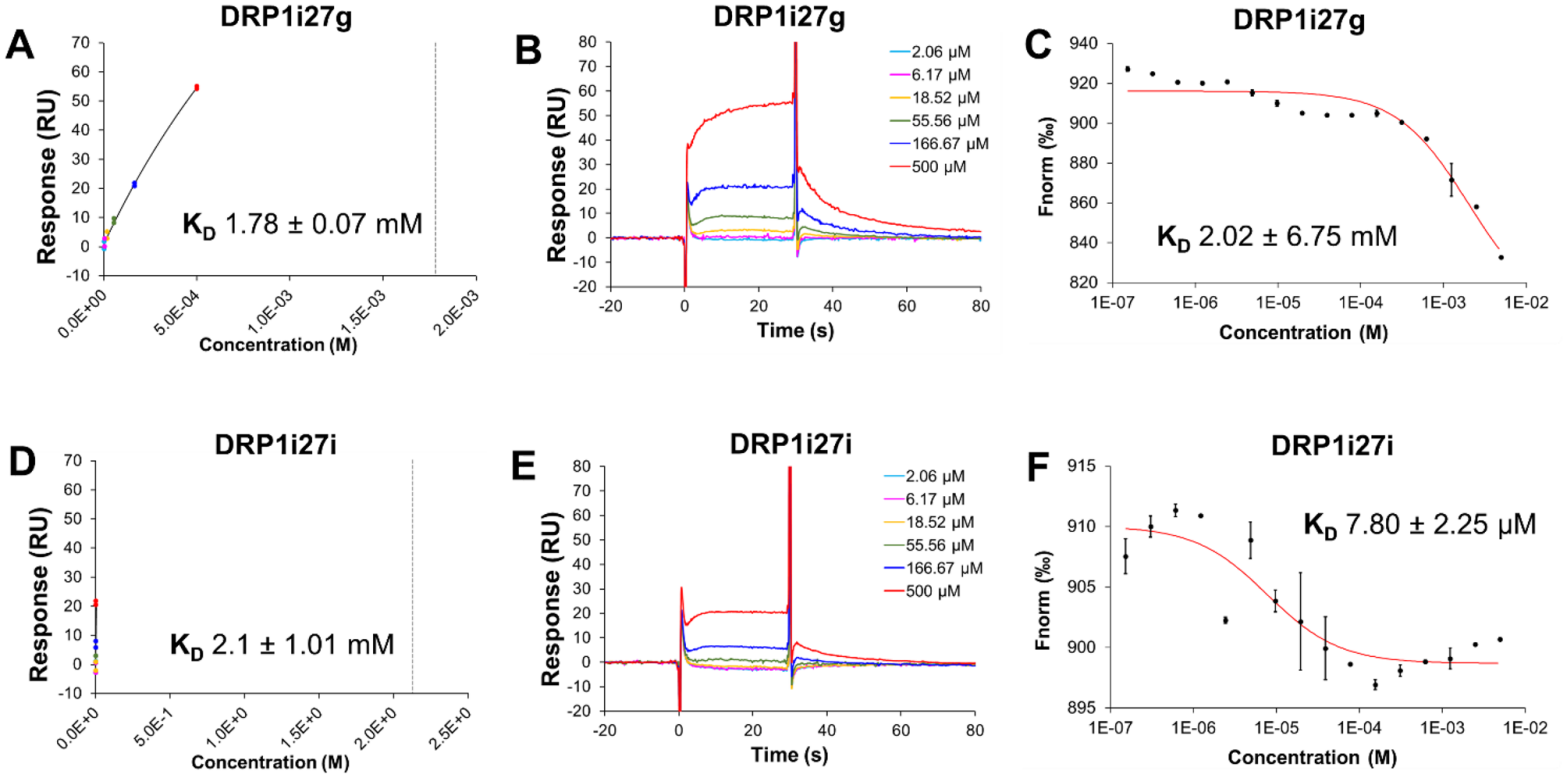
Binding affinity of DRP1i27 analogues toward human Drp1. Representative **(A, B, D, E)** SPR and **(C, F)** MST assay results of **(A–C)** DRP1i27g and **(D–F)** DRP1i27i. (n = 2). Data are expressed as mean ± SEM.

In order to understand the structure–activity relationships of DRP1i27, we conducted a 2D analogue search on DRP1i27. At 80% Tanimoto similarity index, we purchased 26 analogues of DRP1i27, which we screened via SPR, with confirmation of binding via MST (Table 1). Two of the 26 analogues showed dose–response binding affinity to human Drp1 isoform 3 via SPR (DRP1i27g with an extrapolated K_D_ of 1.78 mM and DRP1i27i with an extrapolated K_D_ of 2.1 mM) and MST (DRP1i27g with K_D_ of 2 mM, DRP1i27i with K_D_ of 7.8 µM) (Fig. 7). Notably, both of these compounds were also assayed via SPR in the presence of 20 uM of GppNHp (a non-hydrolysable GTP analogue) resulting in a significant increase in binding affinity for both compounds to Drp1, suggesting these compounds bind in a competitive manner with GTP. Although DRP1i27i showed a promising MST result in particular, overall neither was as active as DRP1i27 and so these analogues were not pursued any further.

**Table 1:** Binding affinity of DRP1i27 analogues to human Drp1 isoform 3. n.b is indicative of no calculated binding.

Id	Structure	SPR	MST	Id	Structure	SPR	MST
		K_D_ mM				K_D_ mM	
a		n.b.	n.b.	b		n.b.	n.b.
c		n.b.	n.b.	d		n.b.	n.b.
e		n.b.	n.b.	f		n.b.	n.b.
g		1.78	2.02	h		n.b.	n.b.
i		2.10	0.008	j		n.b.	n.b.
k		n.b.	n.b.	l		n.b.	n.b.
m		n.b.	n.b.	n		n.b.	n.b.
o		n.b.	n.b.	p		n.b.	n.b.
q		n.b.	n.b.	r		n.b.	n.b.
s		n.b.	n.b.	t		n.b.	n.b.
u		n.b.	n.b.	v		n.b.	n.b.
w		n.b.	n.b.	x		n.b.	n.b.
y		n.b.	n.b.	z		n.b.	n.b.

## Discussion

Drp1 is a major regulator of mitochondrial dynamics and as such is proposed to play an important role in numerous diseases^34^. Despite being the most widely used preclinical Drp1 inhibitor, Mdivi-1 has numerous reported issues including its proposed off-target effects^12–14,35,36^. Mdivi-1 was originally chosen as a lead compound based upon indirect biding assays in yeast whose Drp1 protein construct has < 50% similarity to the human protein. Furthermore, yeast and human Drp1 have been shown to oligomerise differently and have different basal GTPase activity^37,38^.

In our assay buffer containing DMSO and Tween-20, we found Mdivi-1 aggregated at concentrations above 18.5 µM. Colloidal aggregation is a well-known issue in the pharmaceutical drug discovery industry, often leading to false positive results^39,40^. Together, this suggests that some of the biological results interpreted to be Mdivi-1 inhibition of Drp1 may be artifactual and due to Mdivi-1 aggregation. In our experiments, Mdivi-1 showed no binding to human Drp1 via SPR, however showed binding to human Drp1 via MST at a binding affinity much better than expected. MST uses thermophoresis, the movement of molecules through a temperature gradient. Changes to a molecules size, charge or hydration shell are detected as binding of compounds^41^. Combined with the DLS results, this suggests our MST assay was a false positive result, where the reduction in signal may be an artefact of Mdivi-1 aggregation on Drp1. Since our samples were centrifuged before drawing an aliquot for measurement, any aggregates would be removed from the assay before detection and thus this hypothesis seems probable. However, our GTPase assay result does suggest Mdivi-1 may interact with Drp1. Notably, the results of the GTPase assay are likely also confounded by compound aggregation, as no free phosphate is detected above 5 µM of Mdivi-1.

Regardless, it is clear that Mdivi-1 is an unreliable tool compound for human Drp1 inhibition and other inhibitors need to be identified. In recent years there has been some effort to identify novel Drp1 inhibitors, highlighted by two publications reporting 1H-pyrrole-2-carboxamide compounds^42^ and Drpitor1/1a^43^ as the putative Drp1 inhibitors. Notably, neither of these publications describe direct binding of the compounds to the human Drp1 protein via biophysical techniques and only one^43^ shows lack of effect of Drpitorl/1a in Drp1-silenced A549 cells, which are an adenocarcinoma human alveolar basal epithelial cell line.

DRP1i27, identified from a Drp1 virtual screen, is the first compound to date which shows direct binding to human Drp1 via two independent methods. DRP1i27 has a binding affinity of 286 µM in the SPR assay and a K_D_ value of 190 µM via the MST assay. Importantly, DRP1i27 did not aggregate in the assay buffer, which suggests reliability of the protein-based assay results. Although these affinities differ between experiments, this is not unexpected as it well known in the field that no single technique is more reliable than any other and it is important to obtain a positive result across multiple techniques^44^. A good example of why these results could differ is related to the tethering of the protein to an SPR chip which in some cases could occlude the binding site and/or protein dynamics leading to lower affinities. Whereas MST is conducted with protein free in solution however label can occlude essential biding amino acids (i.e. Lys residues). This may also explain the discrepancy between the biophysical assays and the GTPase assay in which we see significantly more inhibition of Drp1 in a label-free, non-tethered environment. Another reason for this discrepancy could be the significantly larger number of moles of DRP1i27 in the GTPase assay compered to Drp1 protein (1 nmol of DRP1i27 compared to 0.0064 nmol of Drp1 protein). Regardless, the activity in this range is typical of an initial hit compound from a screening campaign.

In cells, DRP1i27 increased fused mitochondrial networks of mouse fibroblasts in a Drp1-dependent manner, which was translated in its human counterpart. This effect was also accompanied by cytoprotection against simulated ischaemia–reperfusion injury in HL-1 cells, a mouse-derived atrial tumor cell line. Collectively, these results corroborate previous studies demonstrating the cytoprotective effect of Drp1 inhibition using pharmacological and genetic approaches^2,45–47^.

While none of the tested analogues of DRP1i27 were more active than the parent compound, a dose–response assay conducted with or without 20 uM of GppNHp (a non-hydrolysable GTP analogue) resulting in a significant increase in binding affinity for both DRP1i27i and DRP1i27g compounds to Drp1. This suggests DRP1i27 binds to the GTP binding site of Drp1 in a competitive manner. However, further assays are needed to confirm this. Regardless, these analogues do contribute to our understanding of the regions of DRP1i27 that are important for activity. Molecular docking of DRP1i27 indicates that both ends of the compound are involved in putative hydrogen bonds. Analogues DRP1i27e, DRP1i27k, DRP1i27p, DRP1i27r and DRP1i27u which retain the furan and pyrazole but have an alternative aromatic or heteroaromatic ring instead of the imidazole were found to have no binding affinity. Most of the analogues which retained the imidazole moiety were significantly truncated compared to DRP1i27, with a single aromatic or heteroaromatic ring attached to the diazabicyclic scaffold instead of the furan and pyrazole combination, and thus less likely to be able to bind Gln34 and Asp218 simultaneously. While analogues DRP1i27d, DRP1i27t and DRP1i27z contained an extended aromatic substituent in addition to the imidazole, all were still of clearly different shape and orientation when compared to DRP1i27. For the two analogues which were able to retain some activity, these analogues were smaller than DRP1i27. However, it was interesting to note that the two truncated analogues DRP1i27i and DRP1i27g with binding affinity closest to DRP1i27 both contained a heteroatom in the same position as the furan oxygen of the parent compound while remaining unsubstituted at the adjacent position. This position was essentially unexplored in our 2D analogue search however there are numerous amino acid features in this region which could be utilised to design compounds with greater affinity. Specifically, the side chain of Asp218 forms the edge of this binding site. DRP1i27 is proposed to interact with Asp218 via a hydrogen bond with the pyrazole and this loss may contribute to the reduction of affinity for DRP1i27i and DRP1i27g. This shows exciting promise for future drug discovery campaigns to develop potent Drp1 inhibitors based on DRP1i27 as a lead compound. Due to the modest affinity of DRP1i27, we did not assess to Drp1 over other GTPases. Further lead optimisation will need to address this in the future.

In summary, DRP1i27 is a novel small molecule inhibitor of human Drp1, which exhibited cytoprotective effects in human fibroblasts under oxidative stress, HL-1 cells subjected to simulated ischaemia–reperfusion injury, and human iPSC-derived cardiomyocytes subjected to doxorubicin-induced cytotoxicity. This compound was found to bind directly to human Drp1 isoform 3, with consistent binding affinity via SPR and MST. Molecular docking suggested DRP1i27 bound to the GTPase site of Drp1, with hydrogen bonds to Gln34 and Asp218. The identified hit of DRP1i27 highlights this compound class as novel small molecule inhibitors of Drp1.

## Supplementary Information

Supplementary Information.

## Data Availability

All data is available upon request to Dr Jessica Holien Jessica.holien@rmit.edu.au.
