# Supplementary material for: A novel small molecule inhibitor of human Drp1
Source: Sci Rep. 2022 Dec 13;12:21531. doi: 10.1038/s41598-022-25464-z (PMC9747717; doi:10.1038/s41598-022-25464-z)
Supplement: Supplementary file 1 — Supplementary Information. [file 41598_2022_25464_MOESM1_ESM.docx]

**
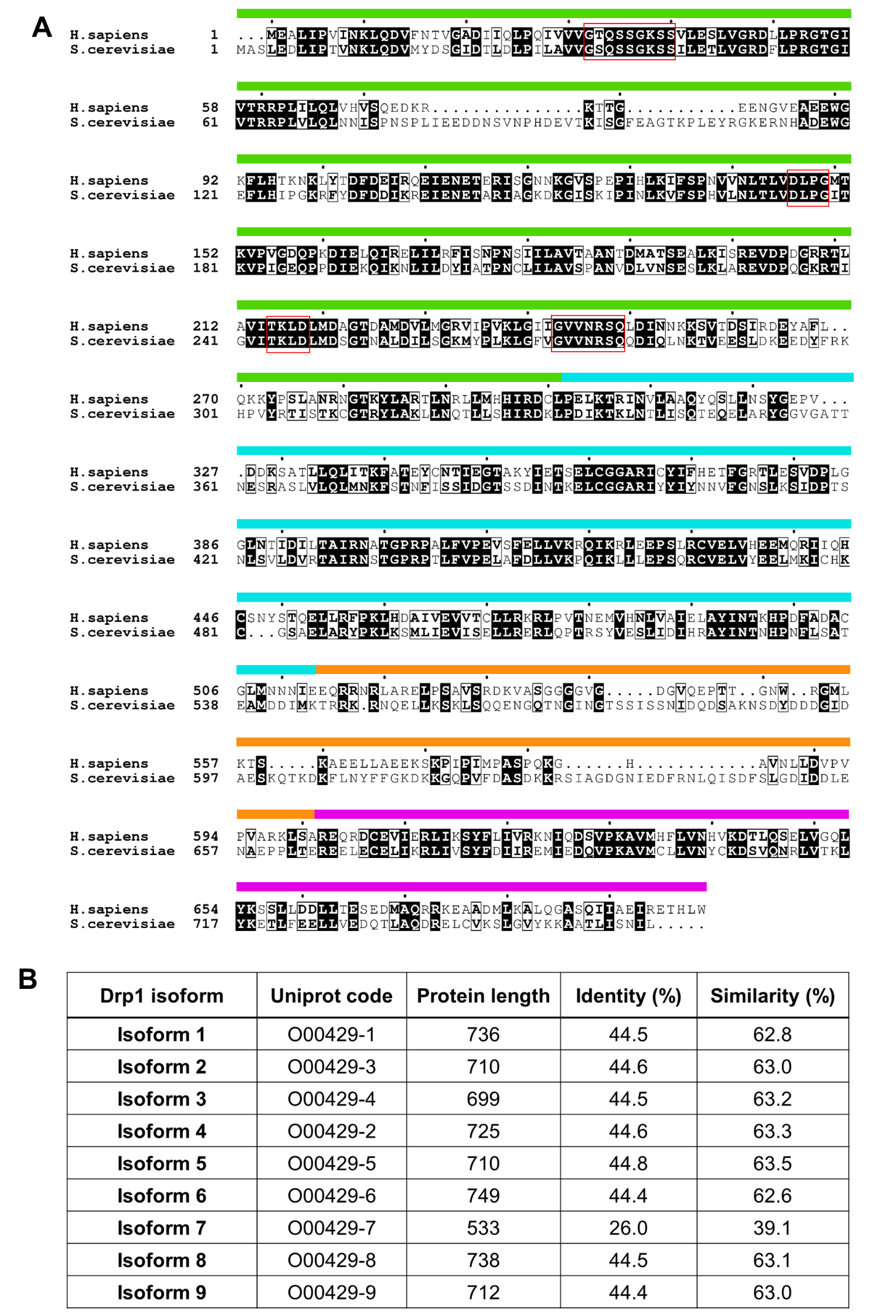
**

**Supplementary Figure 1.** **Alignment between human and yeast Drp1.** **(A)** Representative sequence alignment between human (*Homo sapiens*) Drp1 isoform 3 (O00429-4) and yeast (*Saccharomyces cerevisiae*) Drp1 (P54861) showing 44.5% sequence identity. Green: GTPase domain; Cyan: middle domain; Orange: variable domain; Magenta: GTPase-effector domain; White letters on black background: identical residues; Bold letters: highly similar residues according to physico-chemical properties; Red rectangle; GTP-binding motifs (GTQSSGK, DLPG, TKLD) and a Dynamin-specific G-cap motif (GVVNRSQ). Pairwise global sequence alignment was performed using EMBOSS Needle tool in EMBL-EBI web server. Figure was created in ESPript 3.0 server ([Robert and Gouet, 2014](#_ENREF_28)). **(B)** This is consistent across all isoforms. Human Drp1 isoforms were individually aligned with yeast (*Saccharomyces cerevisiae*) Drp1 (also known as Dnm1; Uniprot code P54861). Pairwise global sequence alignment was performed using EMBOSS Needle tool in EMBL's European Bioinformatics Institute (EMBL-EBI) web server.


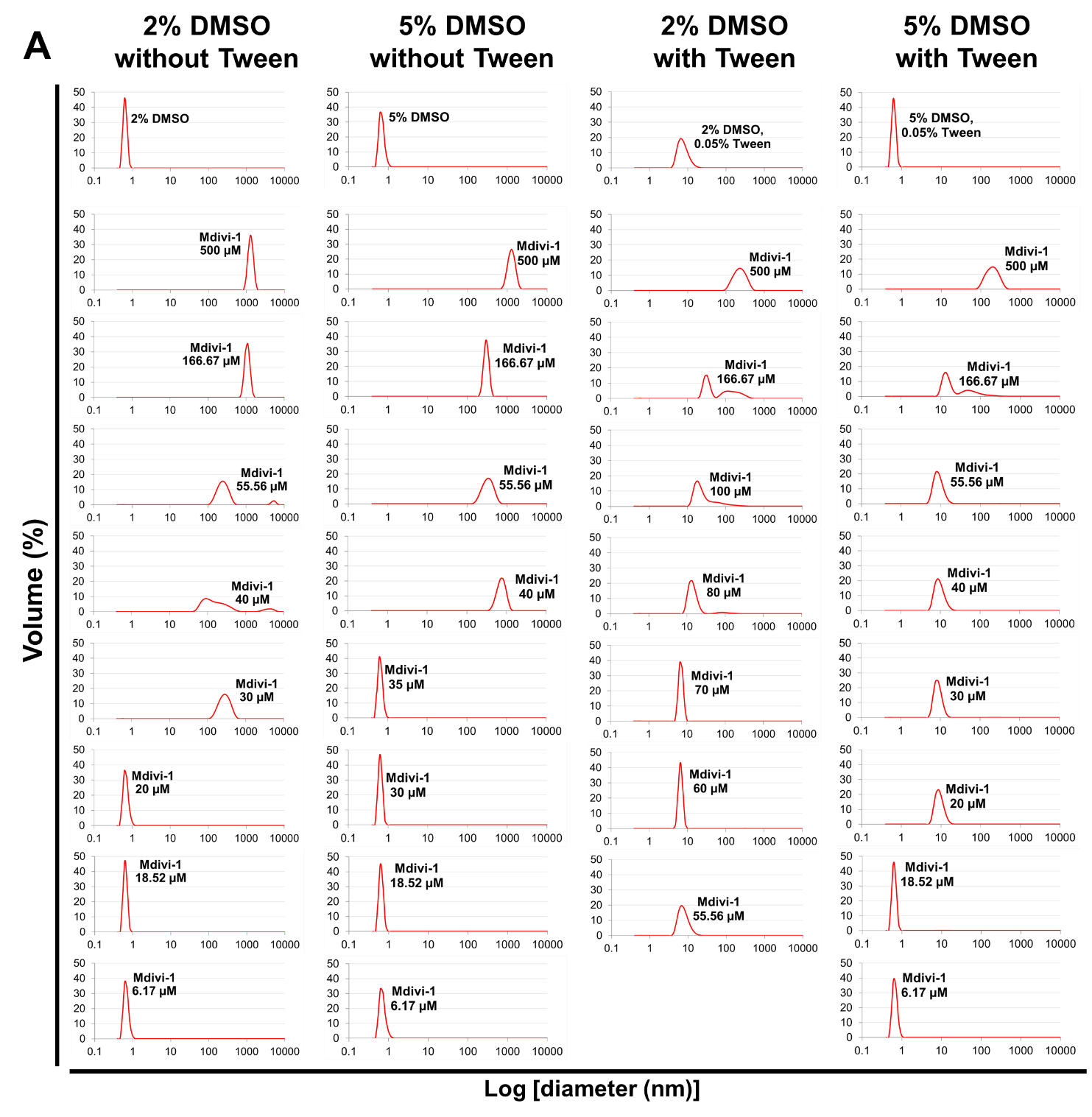


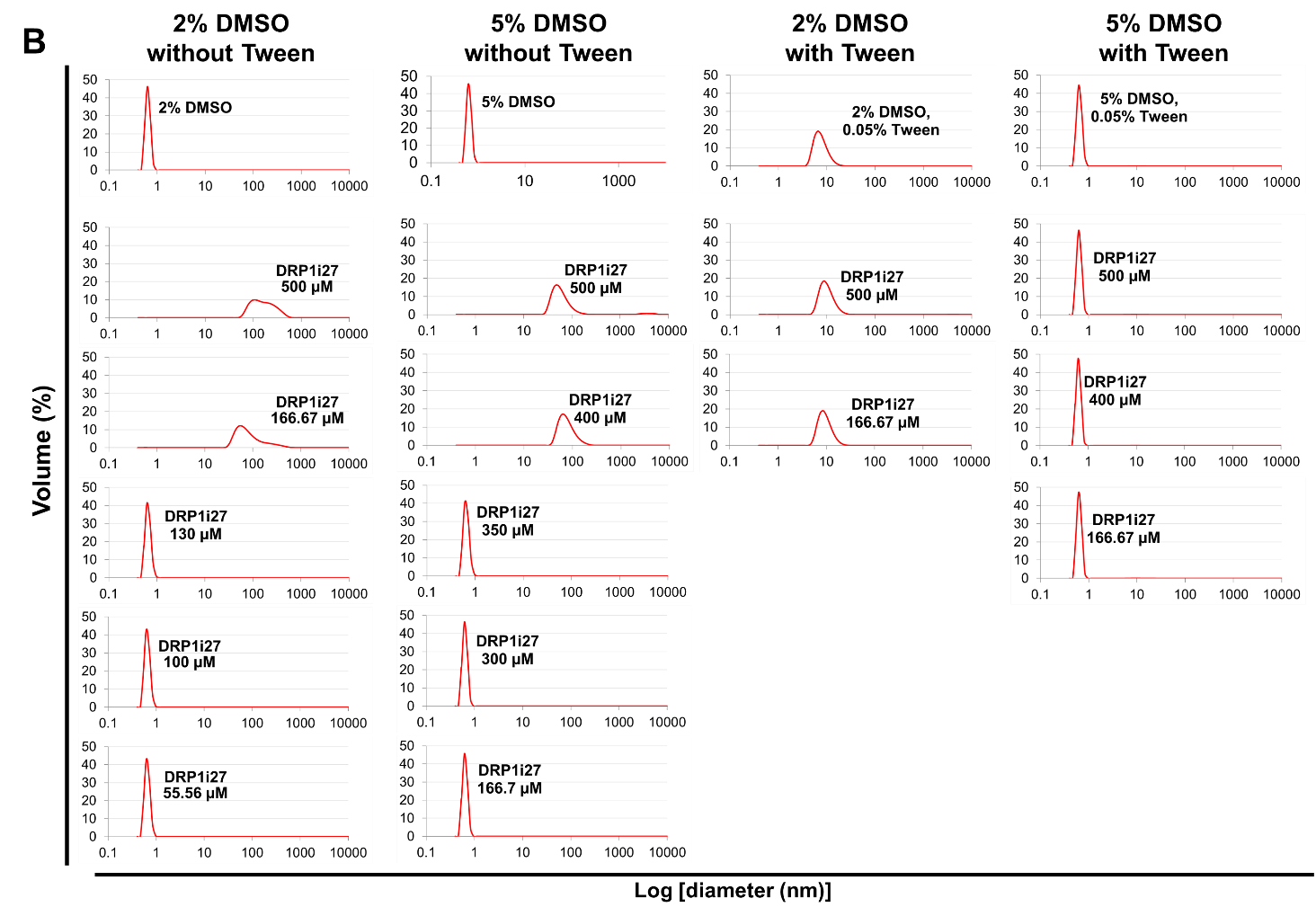


**Supplemental Figure 2. Volume distribution of Mdivi-1 and DRP1i27 aggregates.** Frequency cumulative curves of **(A)** Mdivi-1 and **(B)** DRP1i27 in 2% DMSO or 5% DMSO with and without 0.05% Tween-20.
